# Survey for Highly Pathogenic Avian Influenza from Poultry in Two Northeastern States, Nigeria

**DOI:** 10.1155/2013/531491

**Published:** 2013-07-09

**Authors:** Ibrahim Waziri Musa, Paul Ayuba Abdu, Anthony Kojo Bedu Sackey, Sunday Blessing Oladele

**Affiliations:** Faculty of Veterinary Medicine, Ahmadu Bello University, Zaria, Nigeria

## Abstract

Highly pathogenic avian influenza (HPAI) is a major global zoonosis. It has a complex ecological distribution with almost unpredictable epidemiological features thus placing it topmost in the World Organization for Animal Health list A poultry diseases. Structured questionnaire survey of poultry farmer's knowledge, attitudes, and practices (KAP) in two Nigerian states revealed the presence of risk farming practices that may enable avian influenza high chance of introduction/reintroduction. There existed significant statistical association between farmer's educational levels and AI awareness and zoonotic awareness (*P* < 0.005). Poultry rearing of multiage and species (81%), multiple sources of stock (62%), inadequate dead-bird disposal (71%), and access to live bird markets (LBMs) (62%) constituted major biosecurity threats in these poultry farming communities. Haemagglutination inhibition (HI) test detected antibodies against H5 avian influenza (AI) in 8 of the 400 sera samples; rapid antigen detection test kit (RADTK) was negative for all the 400 cloaca and trachea swabs. These results and other poultry diseases similar to AI observed in this study could invariably affect avian influenza early detection, reporting, and control. We recommend strong policy initiatives towards poultry farmers' attitudinal change and increasing efforts on awareness of the implications of future HPAI outbreaks in Nigeria.

## 1. Introduction

Avian influenza is a highly contagious disease of primarily birds. It is worldwide known to cause devastating effects in poultry to which different strategies ranging from vaccination to stamping out, were employed to control outbreaks in recent past [1–6]. In recent times AI appears to receive most scientific investigations seeking for ways and means of AI virus containment. Notwithstanding, AI has been reemerging with increasing public health impact. In this world without borders to disease spread, no region is protected against a pandemic, and no nation remains safe when all others are at risk of AI incursion [7, 8]. The world is now a global village in terms of international animal trade and movement hence the future wave of pandemic influenza may be difficult to predict. 

Global population growth with increasing levels of poverty and food insecurity seems to initiate changing approaches in agro-livestock practices. The poultry subsector has long been recognised for its potential to significantly contribute to food security and poverty alleviation. As such, it has become so dynamic and highly intensified over the last few decades [8]. This resulted in changes in agro-livestock activities that involved difficulty in controlling trade in live birds, multiple animal species rearing, and paddy rice-fish-pig-poultry integrated farming [9–11]. These mixtures of agro-livestock practices are high-risk-based practices because they are responsible for the maintenance of AI infection. H5N1 HPAI had been reported to thrive in the presence of water bodies, bird faeces, pig populations, domestic and wild water birds, and LBMs [7, 9, 11, 12]. Numerous scientific studies have reported the isolation of avian influenza viruses (AIVs) from surface water at aquatic bird habitats [11]. Live bird markets have sustained and triggered outbreaks and resurgence of HPAI in many parts of the world [11, 13, 14]. Lack of AI knowledge and unacceptable hygienic practices have also led to HPAI infections in animals other than birds [15–18]. It is established that H5N1 HPAI global status is dynamic, and it is also seen to be influenced by virus ecology, and various nations control intervention strategies. This has made future AI epizootics rarely predictable but postulated that next influenza pandemic may likely bear an avian origin [19–21]. This study highlighted the need to continuously evaluate poultry farmer's attitude and practices that may likely affect future early detection and action to be taken with respect to HPAI in northern Nigeria. 

## 2. Materials and Methods

### 2.1. Study Area

The study was carried out in Bauchi and Gombe States in northeastern Nigeria in 2009 and 2011/2012. Bauchi lies between latitudes 10°10′ and 10°33′N and longitudes 9°40′ and 10°13′E in the Sudan Savannah zone [22]. Gombe state is located in the same ecological zone but lies between longitudes 10°45′ and 11°45′N and latitudes 11°15′ and 9°30′E. The people are predominantly farmers. The poultry population is over two million and is made up of mainly rural poultry which are often bought and sold in live bird markets that harbour mixed species of domestic and captive birds [22, 23]. Bauchi State had outbreaks of HPAI in 2006 and 2007 [23, 24]. Despite the closeness and trade in live birds between the two states, Gombe State has not recorded any outbreak of HPAI [23, 25, 26]. 

### 2.2. Sampling Technique

Cluster sampling technique was used to select three local government areas (LGA) from the three senatorial districts in each state, and five villages from selected LGA were randomly selected, making a total of 30 villages used for the study.

### 2.3. Epidemiological Assessment of Risk Factors to HPAI

A total of 170 structured questionnaires on HPAI risk factor assessment were administered to flock owners in various households, commercial poultry farms, and LBMs and where applicable transect walk was conducted for direct observation of poultry premises.

### 2.4. Serological Surveillance for HPAI

About two milliliters of blood was collected through the brachial veins from 200 each of rural and commercial poultry using a 5 mL syringe and a 23-gauge needle. The blood was put in a slanting position and allowed to clot at room temperature overnight. The sera were carefully decanted into 2 mL screw-caped serum tubes (IMEC) obtained from hospital equipment sales outlet in Kaduna, Nigeria. Haemagglutination (HA) and haemagglutination inhibition (HI) tests were conducted using the sera and standard avian influenza H5 obtained from China according to the standard laboratory protocol of OIE (2009). 

### 2.5. Virological Surveillance for HPAI

400 cloaca and trachea swabs were obtained from the birds and tested using Antigen Rapid H5 avian influenza detection test kits obtained from (Animal Genetics diagnostic products) Republic of Korea. The test kit is over 76% sensitive and 100% specific with a detection limit of 10^4.9^ EID_50_/mL or 1 HAU (http://anigene.co.kr/). The test was conducted according to the manufacturer's recommendations. All swab samples subjected to PCR detection were negative.

### 2.6. Statistical Analysis

Data generated from this study were analysed with Chi-square test using Statistical Package for Social Science (SPSS) version 17.5. The *P* value of less than 0.005 was considered significant at 1% degree of freedom. 

## 3. Results

### 3.1. Sample Collection

Table 1 summaries the areas covered, the number of birds sampled and tested during the study. We collected 400 each of tracheal and cloacal swabs and 400 serum samples from commercial and rural poultry. Of the poultry sampled, 40% were from commercial poultry farms, 40% from live-bird markets, and 20% from backyard rural flocks (Table 2).

### 3.2. Rapid Antigen Detection Screening for H5 AIV

Of the 400 each of cloacal and tracheal samples subjected to analysis, 26 were from clinically sick and 374 from apparently healthy commercial and rural poultry. The samples were analysed for the presence of AI type A subtype H5 viral antigens using the rapid antigen detection test kit. Three of the clinically sick birds had clinical features and gross lesions highly suggestive of HPAI (plates 1 and 2). The clinical picture was that of rapid onset of high mortality, severe respiratory distress, cyanosis, and oedema of combs and wattles. Gross lesions observed included petechial haemorrhages in abdominal fats, thoracic cage, cecal tonsils, and proventriculus; trachea and intestines were haemorrhagic and congested. HPAI was initially suspected, and so AI and ND rapid antigen test kits (Anigen Animal Genetics Inc., Republic of Korea) were used on-farm to test for the presence of AI and ND viruses, respectively. The swabs tested positive for ND virus antigen but negative for AI. Samples (whole birds) were taken to AI regional reference laboratory (National Veterinary Research Institute, Vom, Nigeria) for further investigation. ND was further confirmed from the samples sent to the NVRI, Vom, Nigeria. 

### 3.3. Serologic Testing

Since HPAI virus infection lasts a few days in birds and detection of AI virus antigen is best done at the onset of disease particularly during viraemia, virus could have been missed in birds that had the disease before our sampling. Therefore, we conducted serologic screening. Eight of the serum samples were positive for influenza antibodies by HI assay indicating an overall sero-prevalence of 2% in both states. NDV antibodies were detected in 66% (264) of the serum samples. 

### 3.4. Responses from Questionnaire Survey

Most farmers (62.3%) in both states were aware of AI, but only 15.3% were aware of its zoonotic implication (Table 6). Only 9% of farmers could recognise AI disease in poultry (Table 5). The Chi-square statistical test determined significant relationship between education and AI awareness, and recognition and AI zoonotic awareness at 1% degree of freedom with a resultant *P* < 0.005 (Tables 4, 5, and 6). Generally, AI awareness, recognition and zoonotic awareness in the educated group were higher than those in the noneducated groups (Tables 2, 3, and 4). However, recognition of AI was higher in the literates than the illiterates in Bauchi state, while there was no such significant difference in Gombe State.

Multiage birds had the highest responses (81%) indicating their adoption in commercial and rural poultry farming. The multibird species keeping in rural and commercial poultry was observed in 53% of households and commercial poultry farms. Similarly, 53% rural households had other animal species notably dogs, pigs, goats, sheep, and cattle in close proximity to poultry. Also 62% respondents had multiple sources of birds (live bird markets, as gift from friends, and hatcheries) as breeding stocks. On the other hand, hatchery was the sole source of stock for the commercial poultry (Table 7).

Both commercial and rural poultry producers (62%) had relationship with the LBMs in terms of live bird trading. Also, poultry housing in the rural setup was inadequate and where provided was in close proximity to human dwellings. Most commercial poultry farms were not adequately fenced as only 9% respondents had adequate fence. 5% of respondents restricted movement in and out of the commercial poultry farms (traffic control) (Table 7). Sanitary and hygienic conditions of most farms were inadequate as only 7% respondents had good hygienic measures in place (Table 8).

Ponds, streams and wet lands were seen in many rural areas while fish ponds were seen in some commercial poultry farms.

## 4. Discussion

Principally, risk-based veterinary surveillance is aimed at protecting the health of livestock and consumers; to some extent it also allows decision makers to prioritise and allocates resources for effective and efficient disease control strategies. Early diagnosis and rapid response are also essential and critical components for a successful control of highly infectious diseases which are likely to spread rapidly. 

Knowledge, attitude, and practices (KAP) of circumstances that may lead to the introduction, persistence, and spread of HPAI using lessons from worldwide previous outbreaks must be looked into with a view to controlling future possible outbreaks [25]. The KAP analysis of the two states revealed a good level of AI awareness. However, only 9% of farmers could rightly suspect AI occurrence in poultry flocks, and 15.3% of them could appreciate the zoonotic implications of AI outbreaks especially in Bauchi state despite outbreaks of AI in 2006/2007. This appears unacceptable if an early detection and reporting system is to work well. 

The overall low level of AI knowledge in the illiterate group is expected, but it means that advocacy should be intensified using local languages, jingles in the media, organising farmers' forum, and production of self-explanatory graphic pamphlets for farmers. It is worth noting that Gombe State to date had not reported AI outbreak and so the probable reason why AI knowledge appeared not to be significantly different amongst the literate and illiterate farmers. 

Risk-based practices observed in this study included inadequate dead birds' disposal because 84% of farms/households either left birds to rot in the field or fed them to dogs. When birds die as a result of diseases, carcasses remain a source of infection to pen mates and other poultry on the same or other farms. It also means if carcasses are thrown into the field, dogs, cats, and other domestic animals will have access to them. Rodents are normally attracted to such environments and their activities could subsequently contaminate poultry feed and litter materials thereby assisting disease spread and posing great difficulty to disease control. Further, domestic cats have also been found to be naturally and experimentally susceptible to H5N1, and more recently, HPAI H5N1 virus has been identified as a canine pathogen [18, 27–30]. Therefore, deep burial, composting, or burning/incineration of dead or infected depopulated birds, infected poultry litter, and contaminated wastes at the infected sites should be taught to farmers as the recommended approaches to safe disposal of dead birds and infected materials [31, 32]. 

Fish ponds and untreated surface water were observed in some commercial poultry farms and few households during the course of this study. This is because poultry farmers have recently integrated fish farming to poultry. This stands the risk of attracting wild birds especially water fowls that are naturally a source of pathogenic AI. Wild birds were responsible for HPAI H5H3 outbreak in South Africa [33, 34]. Wild birds have also been reported to be infected from infected domestic poultry. 

No poultry farm or household assessed in the study area had complete basic biosecurity measures in practice as indicated in Table 7. It has been documented that contaminated cages, egg flats, feed bags, and personnel played significant roles in HPAI outbreaks where inappropriate sanitary measures and movement restrictions were not imposed between and within farms and households [31]. These high-risk practices also served as major ways of virus spread to and within flocks [35]. It has been recommended that countries should regularly perform risk assessment studies of AI introduction so as to prioritise these risk factors and to find the most appropriate surveillance and control strategies to be applied [31, 36] as the first reported case of Asian type HPAI H5N1 in the African continent was in Nigeria, and to date the definitive route of entry into the country remains debatable [35]. Improved biosecurity and disease awareness have been reported to reduce bird mortality and further limited dissemination of AI within and between flocks [37].

Areas where multispecies and multiage flocks are kept together have assisted in frequent disease transmission and outbreaks [36–38]. Therefore, keeping multispecies, or multiage birds in the same farm premises as seen in 53% and 81%, respectively, of surveyed farms in the study area is risky and dangerous practice. 

Only 41% provided adequate housing for their birds so that poultry had direct contact with wild birds. It has been found out that wild birds are capable of carrying a variety of diseases causing pathogens and parasites.

We found that 62% of farmers in this study (mainly owners of rural poultry) obtained breeding stocks from and sell off their birds at LBMs. Whenever such birds were not sold at the LBMs, they were returned to farms or households together with high risk of infectious agents. Live bird markets have been tagged avenues where viruses tend to travel to and spread out into new areas that had not been previously exposed [24, 39]. In this study 62% of all farmers had access to LBMs. In depth scrutiny showed that sales of poultry at the LBMs were most common with rural poultry owners and a lower percentage of commercial poultry owners were also involved in live bird trading in LBMs. In addition live bird sellers had access to households and poultry farms especially in rural areas to purchase poultry. Some of these people may have previously handled diseased birds, thereby facilitating disease spread into poultry farms. 

As part of the findings of this research, other animals like sheep, goat, cattle, and pigs and other poultry species (ducks, turkeys, geese, quails, and pigeons) were seen to be kept in close proximity to rural and commercial poultry. They could get infected with different AI virus subtypes (especially pigs and quails) which could serve as mixing vessels to enable genetic exchange between different influenza subtypes and a consequent production of a new mutant virus with the ability for a human-to-human transmission. Genetic reassortment with viruses from different species and the emergence of drift variants in a densely human-populated area following all year-round virus circulation are common features of AI [11, 40, 41]. 

## 5. Conclusion and Recommendation 

Risk-based surveillance is used to strategically intervene in operational decisions especially in countries where human and financial resources are limited. Sustainable and sensitive disease surveillance system seems to depend largely on how early and accurately a disease is detected and reported. Farmers' knowledge, attitude, and hygienic practices if enhanced could assist good surveillance and enable timely confirmation of outbreaks by veterinary authorities which will prevent further spread and possible human exposure.

## Figures and Tables

**Table 1:** Selected Local Government Areas, farms, birds sampled and questionnaires administered in Bauchi and Gombe States (2009/2012).

State	LGA	No. of farms/households	No. of quest admin.	No. of birds sampled	No. of sera tested
Bauchi	Bauchi	5	35	70	70
	Katagum	5	25	65	65
	Ningi	5	10	65	65
Gombe	Funakaye	5	25	70	70
	Kaltungo	5	45	65	65
	Y/Deba	5	30	65	65

	Total	30	170	400	400

**Table 2:** Local Government Areas, farms, sera tested, sera positive for AI H5 in Bauchi and Gombe states (*n=400*).

State	LGA	No. of farms/households	No. of sera tested	No. of sera positive
Bauchi	Bauchi	5	70	3
	Katagum	5	65	2
	Misau	5	65	1
Gombe	Funakaye	5	70	0
	Kaltungo	5	65	0
	Y/Deba	5	65	2

	Total	30	400	8

**Table 3:** Literacy level and avian influenza awareness, recognition and zoonotic awareness in Bauchi and Gombe States (2009/2012).

State	AI		AI		AI	
	awareness		recognition		zoonotic awareness	
Bauchi						
Literacy level	Yes	No	Yes	No	Yes	No
Literate	40 (57%)	3 (4.3%)	10 (14%)	33 (47%)	15 (21%)	28 (40%)
Illiterate	6 (8.5%)	21 (30%)	0	27 (39%)	3 (3%)	27 (39%)
Gombe						
Literate	42 (42%)	18 (18%)	15 (15%)	75 (75%)	26 (26%)	34 (34%)
Illiterate	18 (18%)	22 (22%)	0 (0%)	100 (100%)	2 (2%)	74 (74%)

Total	100	100	100	100	100	100

**Table 4:** Chi square tests for literacy level and AI awareness in Bauchi and Gombe States (2009).

State	Lit. level	AI aware	%	AI not aware	%	df	*χ* ^2^	*P*
Bauchi	Literate	40.0	57.1	3.0	4.3	1	36.90	0.000
	Illiterate	6.0	8.6	21.0	30.0	1	36.90	0.000
Gombe	Literate	42.0	42.0	18.0	18.0	1	6.250	0.012
	Illiterate	18.0	18.0	60.0	60.0	1	6.250	0.012

**Table 5:** Chi square tests for literacy level and AI recognition in Bauchi and Gombe States (2009).

State	Lit. level	AI recog.	%	AI not recog.	%	df	*χ* ^2^	*P*
Bauchi	Literate	10.0	14.3	33.0	47.1	1	7.326	0.005
	Illiterate	0.0	0.0	27.0	38.6	1	7.326	0.005
Gombe	Literate	15.0	15.0	45.0	45.0	1	11.765	0.001
	Illiterate	0.0	0.0	40.0	40.0	1	11.765	0.001

**Table 6:** Chi square tests for literacy level and AI zoonotic awareness in Bauchi and Gombe States.

State	Lit. level	AI aware	%	AI not aware	%	df	*χ* ^2^	*P*
Bauchi	Literate	15.0	21.0	28.0	40.0	1	11.99	0.001
	Illiterate	0.0	0.0	27.0	38.6	1	11.99	0.001
Gombe	Literate	26.0	26.0	34.0	34.0	1	17.49	0.000
	Illiterate	2.0	2.0	38.0	38.0	1	17.49	0.00

**Table 7:** Levels of biosecurity practices in 170 farms in the six LGAs of Bauchi and Gombe States (2009).

Biosecurity measures in use	Number positive	Percentage %
Fencing	15	9
Traffic control	9	5
Sanitation and disinfection	12	7
Multiage birds	137	81
Mixed bird species	90	53
Adequate housing provision	69	41
Multiple sources of stock	105	62
Other animals kept with poultry	90	53
Trading in LBMs	105	62

**Table 8:** Hygienic practices in surveyed poultry farms and households in Bauchi and Gombe States (2009/2012).

Sanitation/disinfection	Number positive	Percentage
Dead bird disposal		
Burial	17	10
Incineration/burning	10	6
Left to rot	120	71
Fed to dogs	23	13
Use of protective clothing	15	9
Use of disinfectants	69	41
